# Effect of the Topical Repellent para-Menthane-3,8-diol on Blood Feeding Behavior and Fecundity of the Dengue Virus Vector *Aedes aegypti*

**DOI:** 10.3390/insects9020060

**Published:** 2018-06-04

**Authors:** Jugyeong Lee, Diane B. Choi, Fang Liu, John P. Grieco, Nicole L. Achee

**Affiliations:** 1 Department of Biological Sciences, University of Notre Dame, Notre Dame, IN 46556, USA; jlee58@nd.edu (J.L.); dchoi@alumni.nd.edu (D.B.C.); jgrieco@nd.edu (J.P.G.); 2 Department of Applied and Computational Mathematics and Statistics, University of Notre Dame, Notre Dame, IN 46556, USA; Fang.Liu.131@nd.edu; 3 Eck Institute for Global Health, University of Notre Dame, Notre Dame, IN 46556, USA

**Keywords:** *Aedes aegypti*, repellent, para-menthane-3,8-diol, PMD, blood feeding, fecundity

## Abstract

Dengue fever is an acute disease caused by the dengue virus and transmitted primarily by the mosquito *Aedes aegypti*. The current strategy for dengue prevention is vector control including the use of topical repellents to reduce mosquito biting. Although *N*,*N*-diethyl-m-methylbenzamide (DEET) is the most common active ingredient in topical repellent products, para-menthane-3,8-diol (PMD) is also used commercially. Studies have indicated PMD reduced biting by 90–95% for up to 6–8 h, similar to the efficacy of DEET, depending on the testing environment. The purpose of this study was to evaluate the behavioral effects of PMD on *Ae. aegypti* blood feeding and fecundity to explore the potential impact of PMD on downstream mosquito life-history traits. Two experiments were performed. In both experiments, cohorts of female *Ae. aegypti* (Belize strain) were exposed to 20% PMD or ethanol for 10 min in a closed system and introduced to an artificial membrane feeding system. Following a 30min feed time, mosquitoes of Experiment 1 were killed and weighed as a proxy measure of blood meal, whereas mosquitoes of Experiment 2 were monitored for oviposition, a measure of fecundity. Results showed a statistically significant reduction (*p* < 0.001) in the percentage of *Ae. aegypti* that blood-fed when exposed to PMD (38%) compared to those non-exposed (49%). No significant difference in fecundity between test populations was indicated. These findings suggest that exposure of *Ae. aegypti* to 20% PMD may influence the probability of subsequent blood feeding but of those mosquitoes that do blood feed, egg-lay density is not affected. Further studies are warranted to investigate the full range of effects of PMD exposure on other *Ae. aegypti* life-history traits such as mating, to continue characterizing the potential effects of PMD to impact overall vector population dynamics.

## 1. Introduction

Dengue fever is an acute disease caused by any one of four dengue virus serotypes (DENV1-4) that are transmitted to humans by mosquitoes, mainly *Aedes aegypti*. Almost half of the world, an estimated 390 million people in 128 countries [1], is at risk of infection with DENV [1,2] with associated diseases ranging from asymptomatic to severe dengue (dengue hemorrhagic fever), a potentially fatal outcome [1]. In addition, *Ae. aegypti* is a primary vector of urban Zika virus transmission [3]; an arbovirus of significant public health concern due to neurological disease associated with infection [4]. Although a dengue vaccine, Dengvaxia (CYD-TDV), has been developed by the French pharmaceutical company Sanofi Pasteur, the World Health Organization (WHO) recommends the use of Dengvaxia only in endemic areas with a high density of DENV infections [5]. Likewise, while Zika vaccines are being explored [6], one has not yet been approved for clinical use. Consequently, a primary approach to prevention of dengue and Zika remains vector control [7,8,9].

*Aedes aegypti* feeds predominately during the daytime, with peak biting occurring in the early morning and in the evening before dusk [1], rendering some personal protection (i.e., individual human protection) and vector control measures, such as bed nets, ineffective. Other population-based dengue vector control strategies, such as space spraying with ultra-low volume (ULV) insecticides, thermal fogging or indoor residual spraying (IRS), are used when a quick reduction in the adult *Ae. aegypti* population is needed (i.e., at the beginning of or during an epidemic) [1,2]. However, the effectiveness of IRS has decreased due to insecticide resistance and compliance in affected households [7]. Topical repellents which prevent *Ae. aegypti* biting have also been discussed as an option for dengue control [10]. The gold standard of topical repellents, *N*,*N*-diethyl-m-methylbenzamide (DEET), has been the most common active ingredient in mosquito repellent products for more than 50 years [10]. The efficacy (i.e., prevention of bites on humans) of DEET varies depending on the dosage and vector species affected. There are two competing theories of how DEET works—masking of host odor [10] or confusing the vector receptor [11], an indication that the chemical has a complex physiological effect on the mosquito. Behavioral resistance (desensitization) to DEET has been observed in sequential generations of *Ae. aegypti* [12] and studies exposing same-generation mosquitoes to DEET have shown attenuation of repellent behavior upon secondary exposure [13].

Although DEET currently demonstrates the longest duration efficacy and protection time of any topical repellent active ingredient, it is also a plasticizer with a noxious smell, which influences user compliance [14]. In 2005, the U.S. Centers for Disease Control endorsed the use of topical repellent products containing para-menthane-3,8-diol (PMD), which is derived from the Australian lemon-scented gum tree, *Corymbia citriodora*. PMD is less volatile than essential oils, and thus it can protect a user for a time comparable to that of DEET [15,16]. Because of its natural origin, PMD is perceived as safer by consumers, and because of its “feel” and pleasant scent, it is considered preferable to synthetic repellents [14]. PMD shows similar efficacy to DEET [17] both in the laboratory (90–95% for up to 8 h) and in the field (around 6 h of protection time) at the commercial concentration of 20% PMD compared to 10% and 20% DEET [16]. Despite these findings, research on PMD is far less comprehensive, largely because PMD is a relatively recent discovery. To date, laboratory and field studies of PMD and other plant-derived repellents have concentrated on determining their ability to prevent mosquito bites [14,18] in an attempt to validate novel repellent chemicals [19,20]. Because of the time required in discovery of novel chemicals and development of associated products, it is important to characterize the full range of effects of existing active ingredients on mosquito behavior to better understand their role in reducing the probability of pathogen transmission.

Research of the effects of chemical exposure on *Aedes spp.* vector behaviors other than biting is being explored [21]. Several topical repellent active ingredients, geraniol, eugenol, and citral, have been shown to reduce both host-seeking and blood-feeding behavior of *Aedes albopictus* [21]. Those used as spatial repellents, such as transfluthrin, have been shown to increase *Ae. aegypti* attraction to experimental oviposition sites [22]. In a study by Sugiharto et al. (2016), exposure to 0.14% or 0.16% DEET decreased the size of blood meals taken 3 and 6 h later, respectively [23].

Effect of repellent chemicals on mosquito life-history traits such as blood feeding and fecundity are especially important to observe, as they can have a direct impact on individual mosquito survival and population density, and thus the probability of pathogen transmission. The objective of the current study was to measure the effects of *Ae. aegypti* non-tarsal contact exposure to 20% PMD, the concentration used in commercially available topical repellent products, to characterize the potential effect of PMD on post-exposure behavior associated with human personal protection (blood feeding) and a life-history trait associated with vector population dynamics (fecundity).

## 2. Materials and Methods

### 2.1. Experimental Design

**Figure 1:**
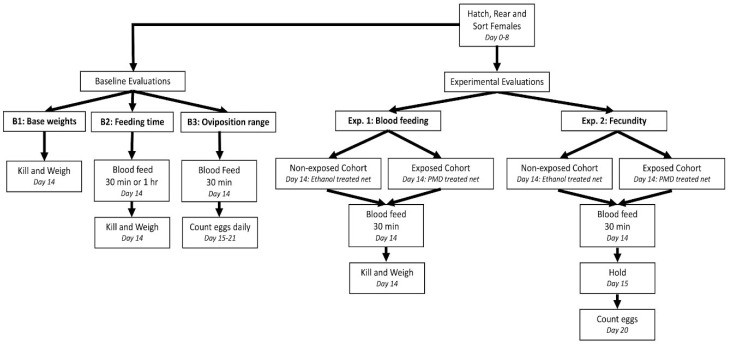
Schematic of study design.

The study consisted of three baseline evaluations and two independent experiments: Experiment 1 measuring *Ae. aegypti* blood feeding behavior and Experiment 2 measuring *Ae. aegypti* fecundity following exposure to either 20% PMD or ethanol (Figure 1). Baseline evaluations were performed to determine the mean variability in weight of non-blood-fed mosquitoes (B1), the optimal blood feeding duration, 30 min or 1 h (B2) and the optimal time period post-blood feeding for monitoring total eggs laid, as a measure of fecundity (B3). For all experimental cohorts, a standardized rearing protocol was used [24] to control for variability in adult size [25], such that any difference in blood meal weight or egg counts could be contributed to a chemical effect.

### 2.2. Mosquito Cohorts

As part of the Ministry of Health, Belize dengue vector surveillance activities, *Ae. aegypti* larvae were collected from Orange Walk Town, Belize in June 2015 and reared to adults at the Belize Vector and Ecology Center (BVEC). Eggs (representing an F1 generation) obtained from these field-collected mosquitoes were used to establish a colony at the University of Notre Dame using previously described protocols [24,26]. All test populations represented 6 day-old females of generations F6–F7.

### 2.3. Chemical Exposure 

**Figure 2:**
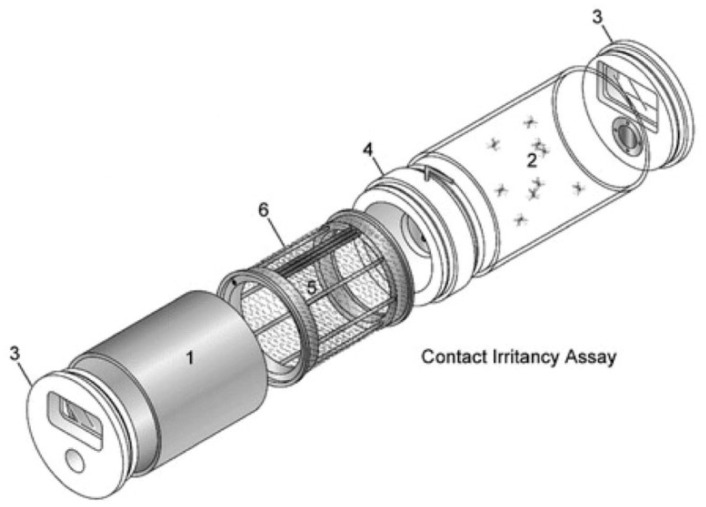
High-Throughput Screening System. Contact irritancy assay systems were used to expose mosquitoes to material treated with either ethanol (control) or 20% para-menthane-3,8-diol (PMD). Test cohorts were prevented from making direct contact with the treated netting. Major components include: 1, Treatment (metal) cylinder; 2, clear (Plexiglas) cylinder; 3, end cap; 4, linking section; 5, treatment drum; and 6, treatment net. Figure used with permission from the *Journal of American Mosquito Control Association* (Grieco et al. 2005).

Female *Ae. aegypti* had access to 10% sugar solution (Instant Nectar Concentrate, Perky-Pet, Lititz, PA, USA) until the age of 6 days and starved for 24 h before blood feeding, with access to water. The cohorts were exposed to either 20% PMD (Takasago International Corp., Crystal Lake, IL, USA), that was diluted in ethanol, (treated) or 100% ethanol (EMD Millipore Corp., Billerica, MA, USA) alone (control) using the contact irritancy assay of the High-Throughput Screening System (HITTS, Figure 2) according to previously described protocols [26,27]. Strips of nylon organdy netting (no. l10N, G-Street Fabrics, Bethesda, MD, USA) were treated with 1.5 mL of solution and allowed to air dry for 15 min, before being positioned in metal chambers of two separate HITSS, one system for PMD treated netting and the other for ethanol treated netting [27]. A clear chamber was connected to each metal chamber by a linking section with a butterfly valve (Figure 2) that was modified to have a piece of untreated mesh over the opening of the valve to prevent test mosquitoes in the clear chamber from making tarsal contact with the treated netting in the metal cylinder (Figure 2). This ensured exposure through the airspace as may occur under field conditions. For each trial, cohorts of 10 mosquitoes were mechanically aspirated into a clear chamber and exposed to PMD or ethanol in separate HITSS simultaneously for 10 min. All exposure assays were conducted at 25 °C and 45% RH. Following exposure, mosquitoes from each HITSS were mechanically removed and held in individually labeled containers at 28 °C and 80% RH until blood feeding, which was conducted within 1 h of HITSS exposure. 

### 2.4. Experiment 1—Blood-Feeding Behavior

**Figure 3:**
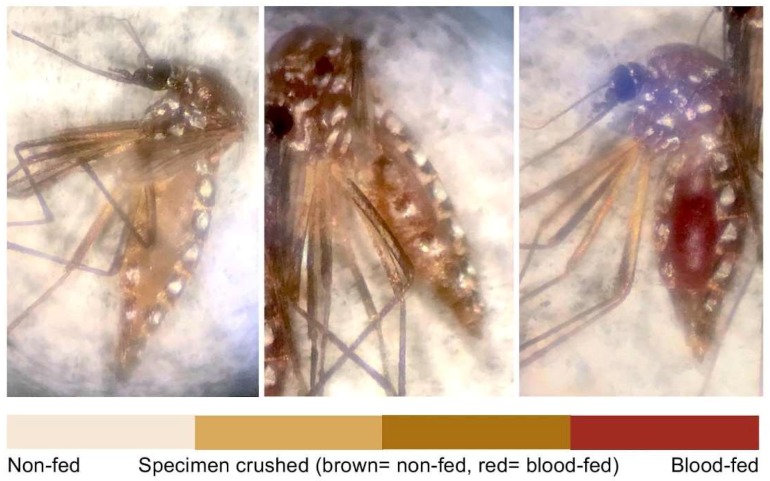
*Ae. aegypti* examination following blood feeding (Experiment 1). Females were categorized as either non-fed (**left**) or blood-fed (**right**) using microscopic observation. For those specimens that could not be distinguished visually, blood-intake was confirmed by crushing the abdomens onto filter paper to observe content color.

The objective of Experiment 1 was to measure the effect of 20% PMD exposure on *Ae. aegypti* blood meal volume using mosquito weight as a proxy indicator. A trial was defined as a day of testing, and a total of 9 trials were conducted. Each trial consisted of 6–10 replicates, each of 20 females, 10 exposed and non-exposed. Females were provided access to human blood (Interstate Blood Bank, Inc. Memphis, TN, USA) containing a 5 mM solution of ATP (Sigma-Aldrich, St. Louis, MO, USA) for 30 min in a temperature and humidity-controlled room (28 °C and 80% RH) using an artificial membrane feeding system according to established protocols [26]. Exposed and unexposed cohorts of each replicate were blood fed simultaneously on separate feeding systems. After blood feeding, test populations were freeze-killed (−20 °C) then weighed. Both exposed and non-exposed cohorts of each replicate in a single trial were blood-fed and freeze-killed at the same time, to control for potential bias in blood digestion (range of holding time until kill amongst trials was 2.5 h). Individual females were weighed, using a digital analytical balance (0.1 mg, AB104, Mettler Toledo, Columbus, OH, USA), and observed for blood meal presence visually by liquid color and abdominal distention (Figure 3). In the cases where the identity of the meal (i.e., blood or sugar-water solution) was uncertain, the abdomen was crushed, and the blood meal was distinguished by the liquid color on the paper.

### 2.5. Experiment 2—Fecundity

The objective of the second trial was to measure the effect of 20% PMD exposure on *Ae. aegypti* fecundity. A total of 4 trials were conducted with each trial consisting of 2–6 replicates. Each replicate contained 20 females, 10 each exposed and non-exposed. After blood feeding, mosquitoes were held for 24 h then individually transferred to single containers with a disposable medicine cup (1 oz., Prime Medical Store, Houston, TX, USA) lined with a 2 cm × 7 cm brown coffee filter (Melitta, Minden, Germany) to serve as an oviposition site. Females were maintained for 5 days with access to a 10% sugar solution renewed daily. On the sixth day after blood feeding (Day 20) mosquitoes were freeze-killed, and total accumulated eggs counted for each female. 

### 2.6. Data Analysis

Tests for normality of data were conducted prior to statistical analyses. The proportions of mosquitoes that blood-fed or oviposited were compared between 20% PMD exposed and non-exposed cohorts, using Chi-square tests (Table 1). For Experiment 1, the data set was divided into two subsets: mosquitoes that blood-fed and mosquitoes that did not blood feed. A two-way ANOVA with factors for exposed vs. non-exposed and blood-fed vs. non-blood-fed, and an interaction between the two factors was conducted to compare the difference in mean (arithmetic) weights of blood-fed mosquitoes that were either exposed or non-exposed. The analysis allowed the correction for any residual sugar meal effect on blood-fed mosquito weight prior to the comparison between the exposed or non-exposed groups. For Experiment 2, as the objective was to compare egg counts between cohorts of those females that laid eggs, a subset of data was used for analysis whereby females that did not lay eggs was excluded. The egg density was compared using the nonparametric Wilcoxon rank sum test. The statistical tests were performed using R (version 3.3.1; the R Foundation for Statistical Computing, Vienna, Austria) and SPSS software (Version 24.0, IBM Corporation, New York, NY, USA).

## 3. Results

### 3.1. Baseline Trials

The mean (±SD) weight of mosquitoes that were neither exposed nor blood-fed according to standard procedures, was 3.17 ± 3.10 mg, suggesting that standard rearing method was successful in minimizing weight variability (B1). There was minimal difference between the mean weight of the mosquitoes that blood-fed for 30 min (3.79 ± 1.23 mg) and the mean weight of the mosquitoes that blood-fed for 1 h (3.89 ± 1.28 mg). Therefore, 30 min blood feed was chosen for subsequent trials (B2). 

**Figure 4:**
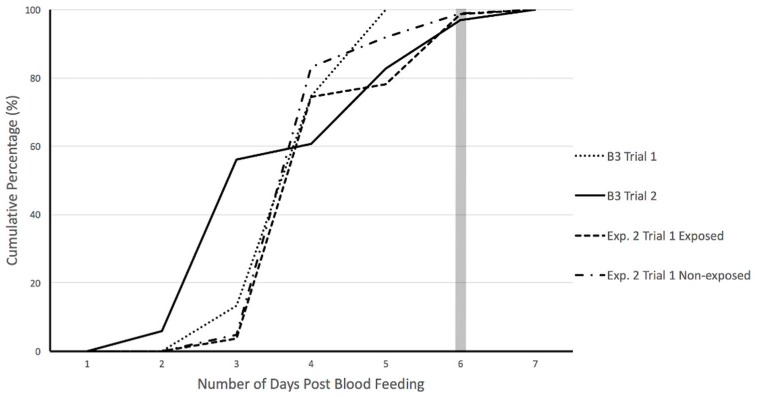
Cumulative percentage of eggs oviposited by female *Ae. aegypti* during baseline and experimental trials. Baseline trials (B3 Trial 1 and 2) were conducted to identify maximum range for egg-lay monitoring. PMD exposed and non-exposed cohorts were monitored for 6 days post-feeding during experimental trials (Exp. 2 Trial 1: exposed and non-exposed).

Oviposition was monitored for individual females every day for up to 7 days post-blood feeding during baseline trials (B3) to determine the number of days beyond which no further egg-laying would occur during fecundity experiments. Results indicate maximum number of eggs were laid upon 6 days post-feeding; after day 6, egg count plateaued (Figure 4). Therefore, egg-laying by individual females following PMD exposure was recorded at one time as an accumulated total at 6 days post-feeding (Day 20).

### 3.2. Experimental Trials

#### 3.2.1. Experiment 1—Blood-Feeding Behavior

The mean (±SEM) percentage of blood-fed mosquitoes that were exposed and non-exposed was 38.1 (±0.8%) and 49.1 (±0.8%), respectively, implying that mosquitoes exposed to PMD were less likely to blood feed than those in the non-exposed (*p*-value < 0.001). PMD does not seem to affect imbibing as the weight, a proxy indicator of blood meal volume, was not statistically different between the two groups.

#### 3.2.2. Experiment 2—Fecundity

The mean (±SEM) percentage of mosquitoes that oviposited was 55.5 (±3.70%) with an average egg count of 62.0 (±4.00) in exposed and 58.9 (±3.67%) with an average egg count of 54.0 (±4.00) in unexposed. (Table 1). Neither the proportion of mosquitoes that oviposited nor the number of eggs produced was statistically different between the exposed and non-exposed mosquitoes (Table 1). 

## 4. Discussion

The objective of the current study was to evaluate the effects of exposure of 20% PMD on *Ae. aegypti* blood feeding and fecundity under laboratory conditions. Although PMD is listed as a topical repellent for mosquito bite prevention [14,15,16], downstream effects on mosquito life-history traits from non-tarsal chemical contact have yet to be exploited [21,22,23]. PMD has similar efficacy to DEET, a gold standard topical repellent currently on the market [16], but unlike DEET, PMD has a natural origin, citrus odor and non-greasy texture following application that may translate into an increase in user compliance [14]. Characterizing the range of effects following PMD exposure is important and may lead to novel uses that exploit changes in mosquito post-exposure blood feeding, blood feeding duration [28], blood meal size, fecundity and subsequent population density, all of which can impact the transmission of arthropod-borne diseases. 

Results of Experiment 1 in the current study indicate that PMD exposure was associated with a significant reduction in the number of *Ae. aegypti* feeding. This downstream effect is especially noteworthy for *Ae. aegypti*, due to the species’ natural blood-feeding behavior to include multiple contacts with a blood source every gonotrophic cycle [28,29]. The possible reduction in mosquito-human interactions by at least one per vector per egg-laying cycle may presumably translate into a reduction in the number of infectious mosquitoes in the environment and thus a decrease in DENV transmission, as vector population and dengue transmission models use biting probability in their calculations [30,31]. Data generated in this and similar studies are anticipated to contribute to parameterization of such computational models. 

As opposed to a reduction in the total number of *Ae. aegypti* feeding following PMD exposure, there was no significant difference in the weight (blood meal intake) of exposed and non-exposed mosquitoes. Since the weights of non-fed specimens in both exposure and baseline experiments were similar, the size variability in test populations was deemed successfully controlled by standardized rearing procedures. Therefore, weight of mosquitoes was a viable proxy indicator of blood intake. This suggests that for those mosquitoes that do feed following PMD exposure, the imbibing mechanism remains similar to those non-exposed. Such results highlight the complexity in mosquito biting, feeding and imbibing behaviors and the specificity of chemical effects on mechanisms underlying each response. 

Blood-feeding behavior has been separated into four stages: activation, orientation, probing, and engorgement [21]. The design of Experiment 1 in the current study did not provide the means to distinguish among activation, orientation, and probing stages, however, data did suggest that PMD had some effect on the probability to feed and not imbibing. These results support findings from other studies characterizing the repellents geraniol, eugenol, and citral, which inhibited activation and orientation stages only, suggesting that these chemicals affect mosquito odorant receptors, and not gustatory receptors of *Ae. aegypti* [21,32]. The lack of influence on the imbibing stage of blood feeding could prove to be advantageous since a decrease in blood meal volume has been correlated with an increase in the number of subsequent feedings [33]. Prior research on DEET exposure and *Ae. aegypti* also demonstrated no significant change in the number of landing and probing, but a significant decrease in blood meal volume [23]. The mechanism underlying the increase in subsequent feeding was shown to be inadequate distention of the anterior abdomen, which would normally inhibit further host-seeking behavior [34]. An increase in mosquito feeding could result in an increase in the rate of DENV transmission, as the number of people bitten by an infectious mosquito may rise [33]. To further characterize the effects of PMD, future studies should be conducted where all four stages of blood-feeding behavior can be evaluated separately. 

Results from Experiment 2 indicated no difference in the proportion of mosquitoes that oviposited following PMD exposure as compared to the unexposed population. Given that there was no effect of PMD exposure on imbibing (weight in exposed and non-exposed cohorts), the similar proportions of egg-laying are not unexpected, i.e., similar blood meal was available for egg development. The trend of increased fecundity (number of eggs laid) in the exposed group in our study differs from that of experimental studies using *Anopheles gambiae* exposed to transfluthrin coils (0.03%), metofluthrin (0.00625%) and DDT (2g a.i. per m^2^) [35]. That study described a decrease in both the number of mosquitoes that fed on humans and the number of eggs laid [35]. However, variability between the two studies most likely is a result of exposure route (HITSS assay vs open-air experimental huts), chemical dosage, and mosquito species. 

The authors recognize several limitations in the current study. First, evaluating the presence of a blood meal was conducted visually, and classified using binary categorization of fed or non-fed, which may not have captured blood meals of minimal volume (i.e., lack of abdominal color change or distention). Increasing categorical analysis should be considered in future studies. Approaches for measuring blood meal volume qualitatively have been described [23,36]. For example, rhodamine B, a florescent red dye, can be incorporated into the blood to mark the females that have blood-fed [37] but novel methods that allow more objective measurement of blood meal are of interest. Second, although air concentration of PMD was not measured during exposure assays, chemical particles were expected to be circulating in the central chamber of the HITSS as PMD is more volatile than DEET, which has been evaluated for non-contact spatial repellent effects in the HITSS with success [27]. In the current study, *Ae. aegypti* that were held for evaluating fecundity were not observed for blood meal intake to control for unintentional killing during handling. Therefore, when the subset of non-zero data points was used for analysis, females that may have blood fed but did not oviposit, a phenomenon known as gonotrophic dissociation [38], could not be identified but which may be of interest. Additionally, egg counts were not recorded daily in Experiment 2 beyond Trial 1, therefore the temporal rate of egg laying was not assessed following PMD exposure. Such an observation scheme could identify potential delays in oviposition due to PMD exposure and should be considered. Lastly, the use of a membrane feeding system limits direct translation of results to the field due to a constrained feeding arena, lack of human odors and CO_2_ cues during feeding, among other biases. Future studies should consider comparing exposure effects of PMD with DEET or other chemicals used in topical repellents. Evaluating the secondary DENV vector *Aedes albopictus* and various *Ae. aegypti* geographical strains against other concentrations of PMD are warranted to validate the generalizability of findings.

In conclusion, PMD is classified as a topical repellent to reduce mosquito biting [14,15,16]; however, results of this study suggest that PMD may also affect other behaviors and life history traits, specifically the feeding probability of *Ae. aegypti* post-exposure. Even though underlying mechanisms of PMD in mosquitoes are currently unknown, results of the current study indicate that non-tarsal exposure may not affect the pathways that drive imbibing or egg development. Characterizing the range of non-contact effects elicited by PMD, and other chemicals used in mosquito control products, is important to the overall effort to prevent human-vector contact and pathogen transmission thereby should be considered in computational models. Such studies will further optimize and guide expectations of existing tools in integrated vector control strategies [39].

## 5. Conclusions

Non-tarsal exposure of *Ae. aegypti* to PMD decreased the number of mosquitoes that blood-fed, but not the size of the blood meal nor the number of eggs oviposited. The demonstrated effects of PMD, which extend beyond its listed use as a topical repellent, warrant further exploration of alternative uses of repellent chemicals on the market to optimize vector control strategies.

## Figures and Tables

**Table 1:** *Ae. aegypti*^1^ (Belize) blood-feeding and oviposition effects following exposure to para-menthane-3,8-diol (PMD).

	Exposed	Non-Exposed	*p*-Value
% blood-fed ^2^ $\bar{x}$ ± SEM	38.1 (±1.84) n = 700	49.1 (±1.89) n = 700	<0.001
Weight blood-fed ^2^ $\bar{x}$. (mg) ± SD	5.24 (±0.91) n = 267	5.31 (±0.96) n = 344	0.2394
Weight non-blood-fed ^2^ $\bar{x}$ (mg) ± SD	2.47 (±0.47) n = 433	2.44 (±0.47) n = 356	
% oviposited ^3^ $\bar{x}$ ± SEM	55.5 (±3.70) n = 180	58.9 (±3.67) n = 180	0.594
No. eggs ^3^ $\bar{x}$ ± SD	62.0 (±4.00) n = 100	54.0 (±4.00) n = 106	0.141

**^1^** F6-7, 6 days old, 24 h sugar-starved, 30 min blood-fed. ^2^ 35 replicates. ^3^ 9 replicates.

## Supplementary Materials

The following are available online at http://www.mdpi.com/2075-4450/9/2/60/s1, Table S1: Baseline—Base weight, Table S2: Baseline-Blood feeding, Table S3: Baseline—Fecundity, Table S4: Experiment 1—Blood feeding, Table S5: Exp. 1—blood-fed only, Table S6: Experiment 2—Fecundity, Table S7: Data Dictionary.
